# Epigenetic Factors and ncRNAs in Testicular Cancer

**DOI:** 10.3390/ijms241512194

**Published:** 2023-07-30

**Authors:** David Nuñez-Corona, Estefania Contreras-Sanzón, Jonathan Puente-Rivera, Rodrigo Arreola, Minerva Camacho-Nuez, José Cruz Santiago, Edgar Antonio Estrella-Parra, Julio César Torres-Romero, César López-Camarillo, María Elizbeth Alvarez-Sánchez

**Affiliations:** 1 Posgrado en Ciencias Genómicas, Universidad Autónoma De México (UACM), San Lorenzo 290, Col. Del Valle, México City 03100, Mexico; 2 División De Investigación, Hospital Juárez De México, México City 07760, Mexico; 3 Departamento De Genética, Instituto Nacional De Psiquiatría “Ramón De la Fuente Muñiz”, Calz. Mexico, Xochimilco 101, Col. Huipulco, Tlalpan, México City 14370, Mexico; 4 Hospital De Especialidades Centro Médico Nacional La Raza, IMSS, México City 02990, Mexico; 5 Laboratorio De Fitoquímica, UBIPRO, FES-Iztacala, Unidad Nacional Autónoma de México, Av. De los Barrios No.1, Los Reyes Iztacala, Tlalnepantla 54090, Mexico; 6 Laboratorio De Bioquímica y Genética Molecular, Facultad De Química, Universidad Autónoma De Yucatán, Calle 43 s/n x Calle 96, Paseo De las Fuentes y 40, Col. Inalambrica, Yucatán 97069, Mexico

**Keywords:** testicular cancer, non-coding RNAs (ncRNAs), epigenetics, gene expression, microRNAs (miRNAs)

## Abstract

Testicular cancer is the most prevalent tumor among males aged 15 to 35, resulting in a significant number of newly diagnosed cases and fatalities annually. Non-coding RNAs (ncRNAs) have emerged as key regulators in various cellular processes and pathologies, including testicular cancer. Their involvement in gene regulation, coding, decoding, and overall gene expression control suggests their potential as targets for alternative treatment approaches for this type of cancer. Furthermore, epigenetic modifications, such as histone modifications, DNA methylation, and the regulation by microRNA (miRNA), have been implicated in testicular tumor progression and treatment response. Epigenetics may also offer critical insights for prognostic evaluation and targeted therapies in patients with testicular germ cell tumors (TGCT). This comprehensive review aims to present the latest discoveries regarding the involvement of some proteins and ncRNAs, mainly miRNAs and lncRNA, in the epigenetic aspect of testicular cancer, emphasizing their relevance in pathogenesis and their potential, given the fact that their specific expression holds promise for prognostic evaluation and targeted therapies.

## 1. Introduction

Testicular cancer, a relatively rare form of cancer, that specifically develops in the male reproductive glands located in the scrotum, primarily affects young men aged 15 to 35, with more than 90% of cases originating from germ cells [1,2]. In the United States alone, the annual cost of different types of testicular cancer is estimated at approximately USD $21.8 million. Despite the shift towards active surveillance treatments and reduced hospitalization, the incidence of testicular cancer continues to rise [3].

Most cases of testicular cancer manifest as germ cell tumors, derived from a common precursor, the carcinoma in situ (CIS) testis, the seminomas (SEM), or non-seminomas (non-SEM). In rare instances, older men present testicular tumors, derived from spermatocytic seminomas (SS), which exhibit distinct phenotypic characteristics from classical seminomas [4,5].

Testicular germ cell tumors (TGCT) are the most common malignancies among men in their teenage and young adult years in occidental industrialized countries with a noticeable incidence increase [1,5]. This type of cancer involves a series of modifications at the epigenetic and genetic levels, transforming the primordial gonocyte [6].

Several risk factors have been identified for the development of testicular cancers such as the cryptorchidism (when one or both testicles fail to descend from the abdomen to the scrotum during fetal development), which increases the risk 2–8-fold [7,8]. Family history also plays a role: men with a first-degree relative with testicular cancer have a four-fold risk increase. Age is another factor, with the highest incidence rates occurring in men aged 15 to 35 [2,9]. A previous history of testicular cancer increases the risk of developing cancer in the other testicle. While the evidence is not entirely consistent, some studies suggest that infertility may be a slight risk factor for testicular cancer [10,11], and there is a potential association between exposure to different factors during adolescence and adulthood such as ethnicity, sexually transmitted infections (STI) such as HPV and HIV, epididymo-orchitis, smoking, and genetic risk factors [12]. While a direct correlation between sexual activity and testicular cancers has not been conclusively established, STIs continue to be the most extensively studied sex-related factor in this context [13].

Promising molecular methods for testicular cancer diagnosis include the analysis of circulating tumor DNA (ctDNA) and liquid biopsy. ctDNA refers to DNA fragments released by tumor cells into the bloodstream, providing information about the tumor’s presence and characteristics. Liquid biopsy involves analyzing various molecules in bodily fluids such as cell-free DNA, circulating tumor cells, serum tumor markers, and circulating microRNAs (miRNAs). These molecular methods hold the potential for accurate and sensitive testicular cancers detection, although further research is necessary to validate and establish their clinical utility, but the miRNAs from clusters 371–373, 302, and 367 are deftly analyzed in liquid biopsy in TGCTs [14].

Non-coding RNAs (ncRNAs) are emerging as important regulators of gene expression in testicular cancers, given their potential as diagnostic and prognostic biomarkers and therapeutic targets. Further research is needed to fully comprehend the roles of ncRNAs in testicular cancers and to develop effective ncRNA-based therapies. Understanding and studying ncRNAs is crucial for determining possible treatments that target these molecules.

In this review, we analyze and discuss current methods of use of testicular cancer biomarkers and diagnosis, exploring the epigenetic factors regulated by ncRNAs, particularly (miRNAs) and long non-coding RNAs (lncRNAs), and highlight the potential they present as biomarkers for disease progression and therapeutic targets.

## 2. Biomarkers in Testicular Cancers: Use in Early Detection, Diagnosis, and Treatment

Early detection of testicular cancer plays a key role in ensuring successful treatment outcomes. Several diagnostic methods are employed, including physical examinations, blood and imaging tests (such as ultrasounds and CT scans), and biopsies (Supplementary Table S1) [12,13,14,15,16,17,18,19,20,21,22,23,24,25,26,27,28,29,30,31,32,33,34,35,36,37,38,39,40,41,42,43,44,45,46,47,48,49,50,51,52,53,54,55,56,57,58].

The integration of various biomarkers, including genomics and proteins, will be necessary to better understand the variability of testicular cancers [59]. A part of this integrative analysis has identified multiple molecular features that can vary between different histologies and reflect the composition of mixed tumors [59]. Testicular tumor biomarkers are mainly found in the spermatic vein more so than in the cubital vein, with positive rates of 89% and 60%, respectively, indicating the presence of circulating tumor markers in all cases of testicular cancer [59,60].

In recent years, molecular methods have emerged as a promising approach to diagnosing that involves analyzing genes, proteins, and other molecules in the body to identify abnormalities associated with testicular cancer. For instance, studies have shown that certain genes such as *OCT3/4*, *NANOG*, and *SOX2* are excessively expressed in testicular cancer cells, and their detection can aid in diagnosis [61].

Various diagnostic techniques have been explored to improve the prognosis and therapy of different testicular cancer types and stages. However, the inconsistency of the results obtained with traditional therapies has emphasized the need to investigate alternative mechanisms to provide more effective treatments. In particular, understanding molecular mechanisms is essential for the development of improved pharmaceutical interventions.

Biomarkers have a crucial role in various clinical aspects in patients with TCGT, including initial diagnosis, prognosis determination, treatment response monitoring, and post-treatment surveillance. Whilst traditional serum tumor markers are essential for clinical management, their limited sensitivity (especially for SEM and teratoma) and the possibility of false positives have led to the exploration of new biomarkers, such as the emerging class of miRNAs [62].

When these biomarkers are obtained from the serum of patients with TGCTs, they exhibit a relatively high predictive power in detecting the presence of tumors. In conjunction with typical clinical presentation, the diagnosis of TGCT can sometimes be made without obtaining tumor tissue. However, the omission of tissue examination in the diagnosis of TGCT should be limited to specific clinical scenarios, where delaying treatment for extremely high-risk patients could significantly compromise outcomes or even result in death. Thus, the initial steps towards the concept of liquid biopsies, aimed at identifying the presence of cancer with minimal invasiveness, were taken well before the emergence of more modern strategies [63].

In this regard, well characterized biomarkers are important for the early detection, diagnosis, and management of testicular cancers. Here are some molecules commonly studied as testicular cancer biomarkers. (1) Alpha-fetoprotein (AFP): A protein produced during fetal development. Elevated AFP levels are commonly used as a diagnostic and monitoring tool for testicular cancer and can also predict the response to chemotherapy in patients [64]. (2) Human chorionic gonadotropin (HCG): HCG is a hormone produced during pregnancy, but elevated levels of HCG are also frequently observed in testicular cancer. HCG levels are often used in combination with AFP to diagnose and monitor testicular cancer. Studies have found that HCG levels can be a significant predictor of relapse in patients with testicular cancer [65,66]. (3) Lactate dehydrogenase (LDH): Elevated levels of LDH are associated with testicular cancer, and this metabolic enzime is often used as a predictor of overall survival in patients [64]. miRNAs: miRNAs are small RNA molecules involved in gene regulation; specific miRNAs are dysregulated in testicular cancer and can serve as biomarkers for the disease. For example, miR-371a-3p is highly expressed in patients with testicular cancer and can be used as a diagnostic biomarker [67].

More than 100 proteins have been identified as cancer-testis antigens (CTA), and with other non-CTA related proteins, these have been proposed as possible biomarkers for testicular cancer. These antigens participate in proliferation, apoptosis resistance, and metastasis; some of them are regulated by DNA methylation, and some are considered oncogenes (Table 1).

## 3. Epigenetic Factors in Testicular Cancer

Epigenetic regulation impacts gene expression without altering the DNA sequence and involves three key components: DNA methylation, histone tail modifications, and non-coding RNAs, recently [78]. DNA methylation involves the addition of a methyl group (CH3) to the CpG dinucleotide sites on DNA, typically found in gene promoter regions, and is facilitated by DNA methyltransferases (DNMTs). Methylation of CpG regions in DNA is associated with gene silencing, as it can hinder transcriptional factor binding or interact with the MeCP2 protein, which recognizes methylated DNA (Methyl-CpG-) [79].

On the other hand, hypomethylation has an opposite effect, leading to gene activation. Methylation not only plays a role in transcription but also influences processes such as X chromosome inactivation, embryonic development, chromatin structure, chromosomal stability, and genomic imprinting [80].

Advancements in the understanding of epigenetic mechanisms, such as DNA methylation, chromatin remodeling, and the regulation of lncRNAs and miRNAs, hold promise for their application in therapy and their use as biomarkers in TGCT. These epigenetic changes have tissue-specific expression and can be detected in bodily fluids such as blood, urine, and semen, enabling non-invasive quantification through liquid biopsy (serum). However, the insights on lncRNA-mediated epigenetic regulation in testicular cancer remain limited, with only a few studies directly linking them to common epigenetic modifications in this type of neoplasm. For instance, levels of demethylation at the 5′ end of the lncRNA *XIST* may serve as a diagnostic marker for testicular cancer. Nevertheless, the potential applications, diagnostic and prognostic, of these lncRNAs in testicular cancer are yet to be fully explored [81].

**Figure 1:**
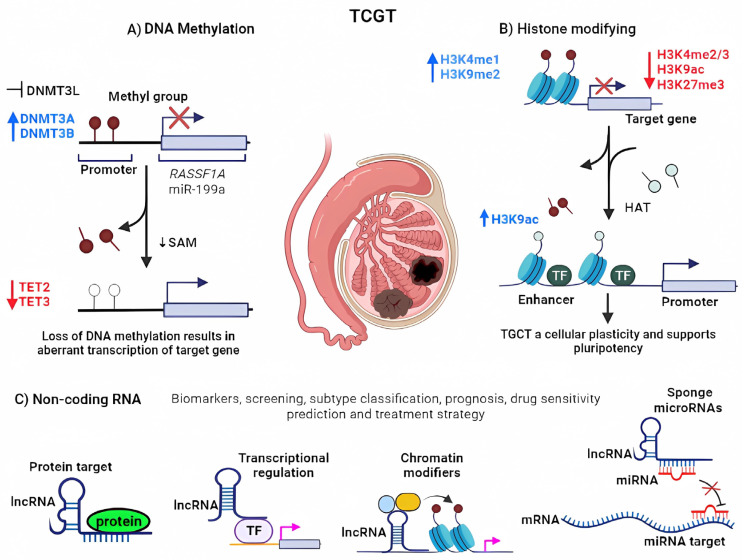
Epigenetic alterations that promote testicular cancer. (**A**) Methylation in promoter DNA or miRNA sequences induces the silencing of gene expression. S-adenosylmethionine (SAM) cosubstrate involved in methyl group transfers. DNMTs activities are up (blue arrow) and down (red arrow) expression in TGCT (**B**) Histone modifying. The removal of the histone methylation marks favors the HATs to acetylate the histone tails, allowing the chromatin to decompress, favoring transcription. Several TGCTs had different methylation or acetylation profiles mediated by up (blue arrows) and down (red arrows) expression of H3K with specific functions as plasticity and pluripotency. H3K4me3 prevents the permanent silencing of genes, perhaps by preventing DNA methylation, whereas H3K27me3 assures that gene expression levels remain low. (**C**) Non-coding RNAs. LncRNAs can act as sponges for miRNAs, scaffold, and silencing tumor suppressor genes and altering chromatin structure. Image created with BioRender.com, accessed on 25 July 2023.

Methylation and acetylation are critical factors that regulate the development of various cancers by modulating gene expression, including the expression of miRNAs (see Figure 1).

### 3.1. DNA Methylation in Testicular Cancers

DNA methylation is the process of covalent addition of methyl groups to cytosine, converting it to 5-methylcytosine (5 mC). It typically occurs in short regions rich in CpG dinucleotides. DNA methylation plays a crucial role in gene regulation, as it can lead to gene silencing when it occurs near or within promoter regions. Aberrant DNA methylation patterns can be observed in cancer, with tumor suppressor genes being silenced through promoter hypermethylation. Testicular cancer is influenced by DNA methylation, and differences in methylation profiles have been observed between SEM and non-SEM, with non-SEM exhibiting greater methylation in tumor suppressor genes [82].

There are three main DNMTs described in mammals. DNMT1 is responsible for conserving DNA methylation patterns during cell division, and its inhibition can lead to abnormalities in spermatogenesis and loss of methylation in paternal imprinting genes. DNMT3A and DNMT3B are involved in de novo DNA methylation and are overexpressed in TGCT [83]. These enzymes are crucial during germ cell development and ensure proper re-establishment of parental imprints [84,85].

On the other hand, DNA demethylases known as TET proteins, particularly TET2 and TET3, have been found to be dysregulated in TCGT [86]. The regulatory factor DNMT3L, which influences DNMT3A and DNMT3B, is downregulated in TGCT [87], indicating the potential impact of dysregulated DNMTs and their regulatory factors on DNA methylation patterns in testicular cancer.

Aberrant de novo methylation of CpG islands in genes related to TGCT is relatively rare compared to malignant testicular lymphomas [88]. However, differences in CpG island methylation have been observed between seminomatous and non-seminomatous germ cell tumors, with SEM showing lower levels of CpG island methylation compared to non-seminomas, with a higher similarity to somatic cancers [89].

Several genes are hypermethylated in testicular cancer, including *APC*, p14 (*ARF*), p16 (*INK*), *GSTP1*, *RASSF1A*, and *PTGS2*. Hypermethylated cell-free serum DNA can be identified in liquid biopsy and could serve as an additional diagnostic parameter in TGCT patients [90]. Methylation of tumor suppressor genes such as *APAF-1* and *DAPK-1* may play a role in TGCT tumorigenesis [91]. *RASSF-1*, located on chromosome 3p21.3, is frequently hypermethylated in TGCT, leading to its silencing and loss of tumor-suppressive function. *RASSF1A* hypermethylation is more common in SEM compared to non-SEM, suggesting its significance in SEM development [92].

**Figure 2:**
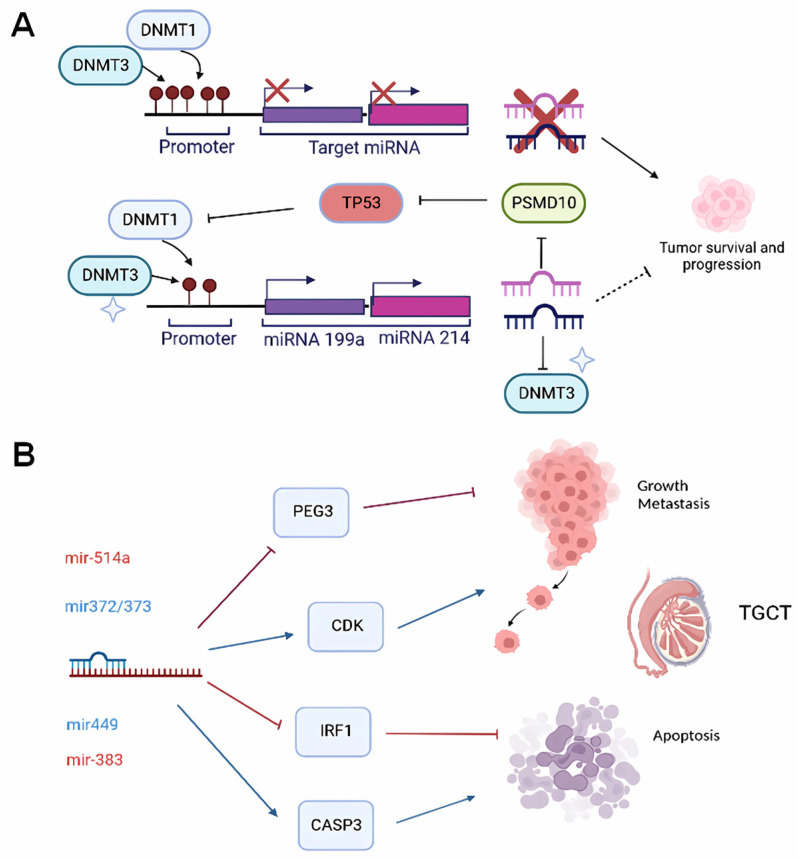
Effect of methylation and function of miRNAs. (**A**) The expression of tumor suppressor genes is regulated by methylation in promoters of miRNAs. The regulatory network of miR-214 (magenta), PSMD10, TP53, and DNMT1 played a role in decreasing the expression of miR-199a and miR-214, in the down-regulation of TP53, and in the increase of DNMT1 in TGCT. miR-199a (purple) also regulates DNMT3. This network partially elucidates the mechanism underlying the DNA hypermethylation of the miR-199a promoter in TGCT and their effect in tumor survival and progression. (**B**) Several miRNAs function as positive regulators (blue font color, blue arrow) or negative regulators (red font color, red arrows) of protein-coding genes involved in cancer hallmarks such as growth, metastasis, and apoptosis and could be potentially used as alternative treatment options. Image created with BioRender.com accessed on 25 July 2023.

The dysregulation of DNA methyltransferases and other enzymes involved in DNA and histone methylation can contribute to the development and progression of testicular cancer by silencing tumor suppressor genes and altering chromatin structure. Further understanding of the specific roles of these enzymes in testicular cancer may pave the way for targeted therapies for this disease (Figure 2).

### 3.2. Epigenetic Modifications of Histones

Gene expression regulation is significantly influenced by the epigenetic modifications of histones. In eukaryotic cells, DNA is packaged by nucleosomes that are composed of octamers of histones (H2A, H2B, H3, and H4). The residues of the N-terminal tails of histones are susceptible to posttranslational modifications (PTM). Some of the modifications that have been described are acetylation, methylation, phosphorylation, ubiquitination, citrullination, formylation, ADPribosylation, lactylation, propionylation, proline isomerization, butyrylation, and crotonylation [93]. Several studies have reported the involvement of histone modifications in TGCT. For instance, the epigenetic state of germ cell carcinomas in situ indicates that low methylation in histones H3K9me2 and H3K27me3 is associated with the repressive state of chromatin, while increased acetylation in H3K9 and methylation in H3K4me and H2A.Z allow gene activation by relaxing chromatin [94].

### 3.3. Methyltransferases

Lysine methyltransferases (KMTs) catalyze the transfer of a methyl group from SAM to the ε-amino group of lysine residues on histone proteins. KMTs regulate chromatin structure and gene expression. Aberrant expression of KMTs has been linked to the development and progression of testicular cancer. KMT is composed of a family of 51 members with a specific domain called SET. Within these families the most prominent subfamilies are Enhancer of zeste homolog 2 (EZH2) MLL, SET, SMYD, SUV, PRDM, and NSD-related proteins, and the members that have shown the highest expression in TGCT are KMT2B, KMT2C, KMT2D, and SET domain-containing protein 1A (SET1A). 

Furthermore, there is another group of methyltransferases responsible for adding methyl groups to arginine residues (PRMT). This family consists of nine members, and the most expressed in TGCT are PRMT8 and PRMT2. Lysine demethylases, which contain a jumonji (jmjC), are dependent on Fe^2+^, and it has been observed that the most altered in testicular cancer are KDM5A and KDM7A [95].

In addition, SET1A is responsible for the mono-, di-, and trimethylation of histone H3 at lysine 4 (H3K4), which is associated with active transcription. Thus, SET1A could be a potential therapeutic target for testicular cancers [96].

EZH2 is a KMT that has been associated with testicular cancer. Its function involves catalyzing a trimethylation of histone H3 at lysine 27 (H3K27), associated with gene silencing. Interestingly, in TGCT, EZH2 is not oncogenic in the malignant transformation and progression events. Instead, the presence of increased levels of EZH2 in normal testicular tissue and low expression levels correlated with the severity of spermatogenic failure suggest that this KMT has potential as a biomarker for defects in sperm production. These findings indicate that EZH2 may have an important physiological role in normal spermatogenesis [97].

### 3.4. Acetylation by Lysine Acetyltransferases (KATs)

Lysine acetyltransferases (KATs) transfer an acetyl group (from acetyl-CoA) to the ε-amino group of lysine residues on histones and other proteins, leading to a more open chromatin structure and increased gene transcription, which plays a significant role in gene expression and epigenetic regulation. On the other hand, lysine deacetylases (KDACs) remove acetyl groups from lysine residues, resulting in a more compact chromatin structure and transcriptional repression, and a dysregulation of KATs/KDACs balance has been correlated to various cancers, including testicular cancers [98]. 

The GNAT (GCN5-related N-acetyltransferase) family includes KAT2A/GCN5 and KAT2B/PCA. KAT6A is upregulated in TGCT compared to normal testicular tissue and influences the expression of genes such as TP53. This gene is involved in cell proliferation and apoptosis. The acetylation of *TP53* by KAT6A may contribute to its degradation and inactivation, leading to enhanced cell proliferation and survival in TGCT [95]. Similarly, KAT2B (PCAF) is overexpressed in TGCT and potentially promotes tumor progression by acetylating and activating the androgen receptor (AR). KAT6A and KAT9 are also overexpressed families in TGCT [96].

To date, the existence of two families of histone acetyltransferases (HATs) has been reported. The MYST family is characterized by having a C2HC zinc finger and an acetyl-CoA binding site, and this family is composed of five members: KAT5 (TIP60/PLIP), KAT6A (MOZ/MYST3), KAT6B (MORF/MYST4), KAT7 (HBO1/MYST2), and KAT8 (MOF/MYST1), and there are two types of histone deacetylases. The first type depends on Zn^2+^ and is categorized into four subclasses: class I (HDAC1, 2, 3, and 8), class IIa (HDAC 4, 5, 7, and 9), class IIb (HDAC 6 and 10), and class IV (HDAC 11) [95]. The second type comprises NAD^+^-dependent Sirtuin deacetylases (SIRTs), also known as class III deacetylases, which include proteins from the SIRT family (SIRT 1–7). Among the most dysregulated deacetylases in TGCT are HDAC9, SIRT2, and SIRT6 [95].

### 3.5. Histone Phosphorylation

Histone phosphorylation is another epigenetic modification that has been implicated in the development and progression of testicular cancers, modulating its interaction with other chromatin components. This dysregulation of histone phosphorylation has been linked to the activation of oncogenes and inactivation of tumor suppressor genes, as well as cell cycle control. In TGCT, a differential expression of ATM and AURKB has been detected between samples of seminomatous and non-seminomatous tumors. The phosphorylation of histones can occur on different amino acid residues, including serine, threonine, and tyrosine, and can be catalyzed by different kinases such as the Aurora kinases, CDKs, and MAPKs [99,100,101,102].

In non-SEM patients, methylation of histone H3 at lysine 9 (H3-K9) results in the suppression of the *RASSF1A* tumor suppressor gene. However, treatment with 5-aza-2′-deoxycytidine (5-aza-dC) reduces methylation in the promoter region of *RASSF1A*, leading to increased gene expression [103]. Conversely, the expression of the *POU5F1* proto-oncogene, which is activated by H3-K9 methylation, has been shown to decrease following treatment with 5-aza-dC [103].

The phosphorylation of histone H3 at serine 10 by Aurora kinase A is associated with the progression of TGCT [103]. Moreover, the phosphorylation of histone H2AX by ATM kinase is also observed in TGCT, with a particularly abnormal and constant presence of pS-ATM in EC, a lesser extent in SEM, and only to a moderate degree in TER [101].

### 3.6. Non-Common Testicular Cancers and Possible Epigenetic Biomarkers

It is also clinically important to identify biomarkers that can predict the effectiveness of genotoxic drugs, so as to avoid exposing nonresponders to potentially harmful treatments. A study utilizing a whole-genome CRISPR/Cas9 gene knockout approach identified ASH2L, a core component of the H3K4 methyl transferase complex, as a protein necessary for bleomycin sensitivity in Hodgkin lymphoma [104]. ASH2L levels could potentially serve as a biomarker for predicting the response to genotoxic drugs in testicular cancer. In cases where tumors express low levels of ASH2L, which may confer resistance to genotoxic treatment, the use of ATR or ATM inhibitors might be more effective, as data suggest that ASH2L knockdown does not affect sensitivity to these inhibitors [104].

Another comprehensive study of 137 primary TGCTs using high-dimensional assays revealed high aneuploidy and a scarcity of somatic mutations. Only three genes, KIT, KRAS, and NRAS, exhibited significant somatic mutations exclusively in samples with SEM components. Integrated analyses identified distinct molecular patterns that characterized the major histologic subtypes of TGCTs, including SEM, EC, YST and TER. Significant differences in global DNA methylation and miRNA expression between histology subtypes highlighted the potential role of epigenomic processes in determining histologic fates in TGCTs. Moreover, a subset of pure SEM with KIT mutations increased immune infiltration and globally demethylated DNA and decreased KRAS’ copy number. The study also reported potential biomarkers for risk stratification, such as miRNAs specifically expressed in TER, and others with molecular diagnostic potential, such as CpH (CpA/CpC/CpT) methylation identifying EC [105].

Primary testicular lymphoma (PTL) is a rare and aggressive disease associated with a poor prognosis. While traditionally reported in patients over 60 years old, it can also occur in younger patients, presenting aggressive, metastatic, and bilateral manifestations at the time of diagnosis, as evidenced in a reported case. Due to its rare incidence and unique behavior, standardized approaches have been challenging. Given its particular tropism for the central nervous system, skin, and contralateral testis, a careful and comprehensive evaluation of patients is necessary, employing a multidisciplinary approach to overcome the difficulties associated with this disease [106].

## 4. Non-Coding RNAs Implicated in Testicular Cancer

ncRNAs constitute a diverse group of RNA molecules that do not encode proteins but play various regulatory roles within the cell. There has been increasing interest in understanding the involvement of ncRNAs in testicular cancers, as their dysregulated expression is associated with disease development and progression [107].

One well-studied class of ncRNAs in cancer is miRNAs, which are small RNA molecules that regulate gene expression at the post-transcriptional level. In testicular cancer, several studies have highlighted the dysregulation of miRNAs, which can act as either oncogenes or tumor suppressors depending on their target genes [107,108]. Notably, the miR-371-3 cluster has shown promise as a biomarker for TGCT [109].

Another class of ncRNAs that have received attention in cancer research is lncRNAs. These large RNA molecules, of around 200 nucleotides (nt) in length, do not encode proteins but instead have diverse regulatory functions within the cell. In testicular cancer, dysregulated lncRNAs have been associated with tumor progression by modulating processes such as cell proliferation, apoptosis, and metastasis [110,111]. One example is the lncRNA HOTTIP, which has been shown to promote testicular cancer progression [112].

Aside from miRNAs and lncRNAs, other types of ncRNAs, including circular RNAs (circRNAs) and small nucleolar RNAs (snoRNAs), have also been implicated in testicular cancer [113,114]. However, the specific functions and mechanisms of these ncRNAs in testicular cancers have not been fully understood, and further investigation is required.

### 4.1. lncRNAs and Testicular Cancers

lncRNAs are RNA transcripts that exceed 200 nt in length. Despite not encoding functional proteins, they perform various biological functions, including acting as transcriptional and post-transcriptional regulators, contributing to structural functions, participating in organelle formation, and maintaining genome integrity [115]. Most lncRNAs are transcribed by RNA polymerase II and possess a 5′ cap and a polyadenylated tail. Classification of lncRNAs is based on their genomic location, which includes intergenic, intronic, antisense, and promoter-associated long RNAs (PALPs) and enhancer-associated RNAs (eRNAs) [116].

In the context of testicular cancers, specific lncRNAs have been identified to play roles in disease progression. For example, lncRNA *SPRY4* has been found to potentially act as an oncogene in TGCT by activating the PI3K/Akt signaling pathway [117]. Table 2 provides a summary of the lncRNAs implicated in testicular cancers.

### 4.2. miRNAs and TCGT

miRNAs are small RNAs (~19 to 24 nt) that act as gene expression posttranscriptional regulators by binding to the 3’ untranslated region (UTR) of target mRNAs. This bind leads to degradation or translational inhibition by posttranscriptional repression of target mRNA, reducing translation efficiency or decreasing mRNA levels. It can produce mRNA destabilization, degradation, and a decrease in protein expression. 

The embryonic stem cell cycle, the proliferation and differentiation of hematopoietic stem cells, and the modulation of inflammation and innate immunity that are regulated by miRNAs have been widely studied in different diseases and linked to the progression in neoplastic processes. These miRNAs can function as tumor suppressors and promote tumor development (oncogenes), or an miRNA can perform both functions on antiproliferative and growth-promoting genes [125,126].

The expression of miRNAs has also been shown to be key in hallmarks of testicular cancer cells such as proliferation, apoptosis, and differentiation, correlated with clinical stage and tumor volume [127,128,129,130,131]. Other deregulated miRNAs have been found in non-SEM tumors and in advanced stages of TGCT. The different participation types of miRNAs in testicular cancer hallmarks are listed in Table 3.

### 4.3. miRNAs Involved in TCGT Progression 

About 20% of testicular cancers patients metastasize after diagnosis. In seminomatous germ cell tumors (SGCT), 62 miRNAs were differentially expressed in tumors with metastasis when compared to tumors without metastasis. Within these, the authors validated three miRNAs: miR-29c-5p, miR-506-3p, and miR371a-5p, confirming their role in metastasis. Therefore, these miRNAs could also be good predictors of metastasis in SGCT [137].

Some of the miRNAs referred to above have been found to be positively related to metastasis. The expression levels of mRNAs are significantly increased in metastatic TGCT patients compared to healthy and non-metastatic individuals; in addition, it was reported that surgical excision induced the normalization of the levels of these miRNAs [136,138,139]. Examples of miRNAs involved in the progresssion of testicular cancer are listed in Table 4.

Given that miRNAs participate in various biological processes, it is vital to know through which pathways and mechanisms the target genes regulated by miRNAs could be modulating such processes and their impact in testicular cancer.

In a set of miRNAs differentially expressed in non-metastatic SEM and metastatic SEM, it was found that miR-99a, miR-125b-2, and let-7a showed statistically significant associations between these groups. In another study, it was reported that the expression of miR-371-73 and miR-302 is not associated with the metastatic state [150]. 

miRNAs have also been reported as potential tumor biomarkers for being more efficient and better at diagnosing, stratifying, and monitoring in comparison with classic tumor biomarkers such as the previously mentioned AFP, LDH, and hCG, that have low sensitivity for the diagnosis and treatment of TGCT. Furthermore, not all patients express these biomarkers, and only 50% of patients will express just one marker. Some biomarkers used, such as LDH, are not specific for testicular cancer [151]. Testicular cancer has a unique miRNA signature; this suggests great value in the diagnosis, prognosis, and treatment of the disease [128].

Serum levels of mIR-371-3 and mIR-302/367 in patients with TCGT were higher when compared to the levels found in healthy individuals, suggesting they are more sensitive and specific to these miRNAs with respect to other tumorous biomarkers. Other miRNAs have been found to be dysregulated in testicular cancer, including miR-17-92 and miR-517a/b [132,152].

### 4.4. Specific miRNAs as a Possible Target for the Testicular Cancers Treatment

miR-200b is a molecule of biological significance that holds potential as a therapeutic target for CAGE-driven cancer by regulating the response to microtubule-targeting drugs [153]. miR-371 has shown higher sensitivity compared to classical biomarkers across all subtypes and clinical stages of TGCT, particularly in SEM at clinical stage 1, which may have important clinical implications [154].

Furthermore, the induction of miR-146a-5p in interstitial Leydig cells exposed to bisphenol A (BPA) has been found to mediate steroidogenic dysfunction by negatively regulating Mta3 signaling and identifies testicular miR-146a-5p as a potential therapeutic target for mitigating the detrimental endocrine effects following BPA exposure [106]. In addition, 13 miRNAs have been proposed for the development of new therapies for TGCT, involving interactions among 31 miRNAs and 13 target genes [2]. miR-371a-3p was specific for TGCT, especially in tracking surveillance after therapy and monitoring residual disease after chemotherapy [136]. Finally, miRNA C19MC and cancer-testis antigens are promising immunotherapy targets in other cancer types such as hepatocellular carcinoma [123] and could also be investigated in testicular cancer.

## 5. miRNAs and Its Epigenetic Regulation in the Testicular Cancer

DNA methylation and histone modifications are epigenetic modifications known to regulate the expression of miRNAs. In TGCT, miR-199a-3p plays an epigenetic role by negatively regulating the expression of DNMT3A at both the RNA and protein levels, inhibiting promoter methylation of APC and MGMT tumor suppressor genes and restoring their expression [87].

The promoter region of miR-199a and miR-214 is methylated by DNMT1, inhibiting their expression in TGCT. miR-199a and miR-214 are expressed together and regulated by the same promoter in TGCT. DNMT1 methylates the promoter region of these miRNAs, inhibiting their expression. miR-214, in turn, increases the expression of the tumor suppressor TP53 by inhibiting PSMD10. The positive expression of TP53 inhibits DNMT1 and increases the transcription of miR-214 and miR-199a [87]. In a cell model study, it was found that hypermethylation negatively regulates miR199a-2, while miR-124a-2 and miR-184 were shown to be up-regulated in TGCT [155]. 

Batool et al. reported that miR-125b is repressed in TGCT by DNA methylation and histone modification. Overexpression of miR-125b promoted its anticancer functions by decreasing the abundance of tumor-associated macrophages (TAM) by regulating chemokine derived from tumor cells CSF1 and CX3CL1 [155,156] 

Whole-genome DNA methylation profiling in TGCT cell lines revealed an inverse correlation between the level of methylation and miR-371/2/3 expression. In addition, the expression of class I HDACs showed differences between SEM and non-SEM [137,157]. In response to E2F1, the expression of miR-449a and miR-34a/b increased, and these miRNAs have tumor suppressor functions by reducing proliferation and promoting apoptosis through p53. On the other hand, the acetylation of p53 at residue Lys 382 is increased by miR-449a and miR-34a/b, which negatively regulate CDK6 mRNA and Sirt1 deacetylase [158,159].

## 6. Strategies for Targeted Therapy Based on TGCT Epigenetics

Targeted therapy strategies for treating TGCT by epigenetic regulation, such as the use of DNA methyltransferase inhibitors in non-SGCT, could reprogram the epigenome to a hypomethylated state and induce immunogenicity [159].

Recent therapeutic treatments have also emerged as alternative options, with the exploration of new drugs such as 5-Aza-2’-deoxycytidine (5-AZA-CdR or DAC), a DNA methyltransferases inhibitor, which has induced delayed tumor growth in murine squamous cell carcinoma SCCVII in C3H/HeN mice [160] and has also shown activity toward cancer-testis antigens [161].

Additionally, CD19-specific CAR-T cell therapy has been shown to be a safe and effective treatment option for patients with testicular relapse with B-cell acute lymphoblastic leukemia [162]. MK-2206, a selective allosteric AKT inhibitor used for the treatment of solid tumors, has also shown induced cytotoxicity and apoptosis in testicular cancer [163]. The T cell receptor from the interaction of MAGE-4 with peptide-human leukocyte antigen Trp-167, which acts as a tunable gateway, is an emerging field of TCR-based therapeutics [164].

Other agents, such as cancer vaccine delivery as nanoparticles with cancer-testis antigens in mouse models, have shown a significant increase in specific IFN-γ frequencies as well as elevated lysis activity toward a target cell line (A375) [165]. However, antitumoral necrosis factor alpha (anti-TNFα) agents such as infliximab, which is an anti-inflammatory drug, are counterproductive in patients with testicular cancer [166]. Moreover, pembrolizumab is well tolerated by patients with TGCT but did not benefit patients with refractory TGCT as shown in cohort studies [57].

Finally, the application of an algorithm for classifying low HCG levels may help to avoid unnecessary treatment in patients with testicular cancers [167].

## 7. Conclusions

In this review, we summarize the significant progress in understanding the role of epigenetic mechanisms in the development and progression of testicular cancer. Specifically, DNA methylation, histone modifications, and ncRNAs have emerged as key players in the disease. While some non-invasive biomarkers have been identified for diagnosing testicular cancer, they often lack sensitivity and precision. Therefore, it is imperative to continue exploring novel markers with higher accuracy to improve diagnosis and develop more tailored and effective treatments for patients with TGCT. ncRNAs hold great potential for elucidating epigenetic regulation in TGCT and understanding the intricate mechanisms of epigenetic regulation, with which we can advance our ability to diagnose and treat this disease more effectively. We believe that ncRNAs may provide researchers with a deeper insight on epigenetic mechanisms in testicular cancer. Furthermore, the study of epigenetic regulation can contribute to the diagnosis and development of more effective therapies.

There are ongoing efforts to propose and evaluate diagnostic prediction models using a combination of biomarkers, particularly miRNAs and lncRNAs. These non-coding RNA molecules have shown great potential as diagnostic markers due to their stable presence in various body fluids and their specific expression patterns in different diseases, including cancer. Combining multiple biomarkers, such as miRNAs and lncRNAs, can enhance the accuracy and reliability of diagnostic predictions by capturing a broader spectrum of disease-related alterations. Integrating these biomarkers into comprehensive diagnostic models holds promise for improving early detection, diagnosis, and personalized treatment strategies, for example, the miR-371a-3p cluster levels as discriminative diagnostic tool in different types of testicular cancers and assessing the presence of metastasis and monitoring treatment success in cisplatin-treated TGCT patients [132,152], and the miR-519/517 cluster could be used as tumor biomarker for advanced-TCGT stage, non-SEM tumors and treatment as tumor resection [167]. However, further research is needed to validate the performance and clinical utility of these models in larger cohorts and diverse populations. Overall, the exploration of diagnostic prediction models based on a combination of miRNAs and lncRNAs represents a compelling avenue for advancing precision medicine and improving patient outcomes.

## Figures and Tables

**Table 1:** Proteins and identified cancer testis antigens (CTA) as posible testicular cancers biomarkers.

Protein	Expression in Testicular Cancers	Function	Reference
MAGEC2	Nucleous, expression increase in seminomas, spermatocytic seminoma (SEM), and intratubular germ cell neoplasia unclassified	Oncogenic activation of the MAPK pathway and impairment of the p53 transactivation function	[68,69]
CAGE	Increased in cell line	AP-1-and E2F-dependent expression of cyclins D1 and E and cell cycle progression	[70]
MAGEA3, MAGEA4, MAGEC1, GAGE1, and CTAG1B	Decreased expression in n intratubular germ cell neoplasia, re-expression in seminomas in contrast to SEM	Loss of cancer testis antigens in early tumorigenesis of TGCT and later re-expression in a subset of SEM	[69]
PRAME	Preferential expression in SEM or within the seminomatous component of mixed TGCT, focal and variable expression in yolk salc tumors (YST) and choriocarcinomas (CC), and no expression detected in embryonal carcinomas (EC) and teratomes (TER).	Repressor of the retinoic acid receptor (RAR) chromatin regulation	[71]
NY-ESO-1	Specific expression in carcinomas in situ and spermatocytic seminomas	Testicular antigenic protein, transcriptional regulation, recruiting histone deacetylase HDAC1	[72]
TOPOII,	Expressed in EC, YST, seminoma and CC of TGCTs, negative expression in TER	Testicular antigenic protein, regulation of pluripotency suppressing somatic/germ cell differentiation in SEM	[73]
GST-M3 protein	Low expression in seminoma, prediction of a higher risk of TGCT	Glutathione S-transferase	[74]
p21-activated kinase 4	Overexpression in EC	Apoptosis resitance	[75]
PIWIL1	Only expressed in TGCT	Specific protein, DNA methylation, and RNA silencing	[76]
CDK10	Expressed in SEM but not in normal tissue	Cell cycle regulation	[77]

**Table 2:** lncRNAs found in testicular cancers.

Name	Class	Location	Property	Pathway	Reference
*SPRY4-IT1* ↑	Intronic	chr5:1423176 20-142318322	Oncogenic properties	MAPK/ERK, PI3/AKT	[117]
*NLC1-C* ↓	Intergenic	chr21:449992 08-45004727	Tumor suppressive	Nucleolin-miR320a/miR-383NLC1-C	[118]
*H19* ↑	Intergenic	chr11:199513 0-2001710	Oncogen/tumor suppressive	PI3K/AKTmTOr pathway	[119]
*HOTTIP* ↑	Antisense	chr7:2719857 5-27207259	Oncogenic properties	ceRNA HOTTI P-miR-1283p/HOXA13	[120]
*THOR* ↓	Intergenic	hr2:11813212 8-118186456	Oncogenic properties	IGF2BP1	[121]
*LIN28B-AS1* ↑	Antisense	chr6:1048644 64-104941447	Oncogenic properties	Cell cycle, IGF2BP1, LIN28B, CCN D2, FMN2, CDKN2A	[122]
*PCAT6* ↑	Intergenic	chr1:2028108 68-202831446	Oncogenic properties	Gametogenesis -related pathways	[123]
*LINC00467* ↑	Intergenic	chr1:2113827 55-211444093	Oncogenic properties	AKT pathway	[124]
*XIST*	Intergenic	chrX:7381777 5-73852753	Inactive X chromosome	Unknown pathway	[112]

↑ High expression, ↓ Low expression.

**Table 3:** Role of miRNAs in testicular cancer hallmarks.

miRNA	Expression	Effect on Cancer Hallmarks	Reference
miR-517/miR-519a	Increased	Migration, invasion, and poor overall survival	[132,133]
miR-383	Increased	Apoptosis, proliferation, and cell cycle regulations	
miR-223-3p	Increased	Migration, invasion, and apoptosis	
miR-449	Decreased	Cell cycle progression	
let-7a miR-26a	Decreased	Cell growth and mobility	
miR-200c-3p	Decreased	Tumor progression	[134]
miR-25-3p	Increased		
miR-302a-3p	Decrease		
miR-367-3p	Increased		
miR-519d-3p	Increased		
miR-96-5p	Increased		
miR-661	Decreased	Invasion	[135]
miR-640	Decreased		
miR-665	Decreased	Invasion	
miR-1204	Increased	Cell proliferation and cell division	
miR-1203	Decreased	Tumor relapse	
miR-650	Decreased	Cell growth and invasion	
miR-1182	Decreased	Proliferation and invasion	
miR-367-3p, 371a-3p, 372-3p and 373-3p	Increased	Correlated with stage and metastasis	[136]

**Table 4:** Differential expression of miRNAs involved in the progression of testicular cancer.

miRNA	Expression	Target	Cellular Effect	Reference
miR-367-3p	Increased (↑)	MDM2	Cell invasion	[140]
miR-373-3p	Increased (↑)	p53	Oncogenic stress	[141]
miR-371a-3p	Increased (↑)	PTEN	Proliferation and metastasis	[142,143]
miR-506-3p	Decreased (↓)	GALNT4 TGF-β1 EZH2	Metabolism of proteins and O-linked glycosilation of mucinsProliferation, migration and invasion, tumor growth, and metastasis in SGCT	[144,145]
miR-371a-5p	Increased (↑)	SRCIN1	Proliferation and metastasis	[146,147]
miR-223-3p	Increased (↑)	CDH6, SHOX2	Metastasis and proliferation	[148,149]

↑ High expression ↓ Low expression.

## Data Availability

Not applicable.

## Supplementary Materials

The following supporting information can be downloaded at: https://www.mdpi.com/article/10.3390/ijms241512194/s1.
