# Supplementary material for: Epigenetic Factors and ncRNAs in Testicular Cancer
Source: Int J Mol Sci. 2023 Jul 30;24(15):12194. doi: 10.3390/ijms241512194 (PMC10418327; doi:10.3390/ijms241512194)
Supplement: Supplementary file 1 [file ijms-24-12194-s001.zip › ijms-2503538-supplementary.pdf]

Supplementary Table S1. Detection methods, treatment and observed results in testicular cancer.

Type of cancer	Detection method	Therapy/treatment	Results/conclusions	Ref.
Testicular teratoma of 63 men.	Physical examination	Radium and the X-ray (8000 mc and 140-200 kilo-volt)	X-ray possess advantages over the radium pack	[15]
Testicular teratoma of four men.	Physical examination	Radium (9,000-20,000 mc) X-ray: 320 ma (8000 mc, 140-200 Kv)	Teratoma recovery	[16]
Testicular teratoma of 11 men.	Histopathology, radioimmunoassay, carcinoembryonic antigen	Vinblastine 10 mg, actinomycin D 2 mg, Methotrexate 200 mg, Folic acid, Bleomycin 15 mg	Destruction of the cells of origin by chemotherapy	[17]
Testicular seminoma of 53 men.	Clinical examination, HCG, immunoassay	Orchiectomy, cobalt-60 or 10 MeV linear accelerator. (2000-5000 rad tumor)	Survival (94%).	[18]
Testicular seminoma of 73 men.	Chemoteraphy/phropilatic irradiation (1500-4000 rad)	Orchidectomy, radiotherapy (175-3000 cGy)	Survival (91%)	[19]
Testicular tumors of 12 men.	Immunoassay	Orchiectomy and radiotherapy (1820-4500 rad)	Induced significant 76% damage	[20]
NSGCT: Stage II. 65 men	Histology-marker	Vinblastine 7.5 mg/m^2, bleomycin I mg, irradiation 4500-5500 rad,	Eliminate recurrence and disease related mortality.	[21]
Testicular GCT of 838 men.	Clinical examination	Orchidectomy, radiotherapy, orthovoltage X-rays, ^{60}Co X-ray	No increase in survival	[22]
Testicular seminoma of 188 men.	Clinical examination, chest X ray	Orchidectomy, radiation therapy (3000 cGy)	Survival (89.5%).	[23]
Stage I testicular teratoma of 248 men.	Clinical examination, chest X-ray, lymphography, urography.	Radiation 40 cGy	Increase the risk of peptic ulceration.	[24]
Metastatic testis tumors of 15 men.	GPx activity, selenium	Orchiectomy, PVB (10 mg, 0.2 mg/kg, 30 mg), mannitol frusemide 10 mg, chlorpromazine 25 mg.	Retention of cis-platin in tissues and subsequent alteration of selenium metabolism.	[25]
Testicular seminoma of 45	Clinical examination	Orchiectomy,	Survival (near 100%)	[26]

men.		orthovoltage 3000 rads		
Testicular metastatic and chemotherapy refractory GCT of 24 men	Biomarkers (HCG, AFP, LDH)	Clophosphamide, cisplatin, doxorubicin, vinblastine, bleomycin	The efficacy of salvage therapy need to identify others variables	[27]
Testicular seminoma stage I of 27 men:	CT scanning, LDH elevated	Orchidectomy, irradiation (2500-3000 cGy)	Irradiating only of para-aortic region (early stage seminoma)	[28]
Testicular semininoma of 128 men.	Histology, clinical examination	Post-orchidectomy radiation therapy (35 cGy)	No tumor related deaths	[29]
121 men: NSGCT of the testis	Histological evidence of markers	CEB	Independent-toxicity of tumor response to carboplatin	[30]
Metastatic testicular stromal cell tumor found in one man	Ultrasound, chest x-ray, AFP and bHCG levels	VIP (75 mg/m^2, 20 mg/m^2, 1 g/m^2)	Complete clinical remission	[31]
Testicular seminoma of 128 men.	Histology	Orchidectomy, irradiation (2500-3400 cGy)	Optimal treatment for patients with testicular seminoma.	[32]
Testicular biopsies of 46 men.	AgNOR staining method	Not treatment	Reliable way to diagnose	[33]
Testicular seminoma of 859 men.	Histology	Radiotherapy	Not risk of mortality secondary related to radiotherapy	[34]
Testicular tumor testis found in one man.	Histological examination, serum hormone levels	POMB with actinomycin D, cyclophosphamide and etoposide	Without evidence of tumor recurrence.	[35]
Risk metastatic NSGCT found in 75 men	Physical examination, levels of AFP, HCG, bHCG, CT (thorax, abdomen and pelvis)	BEP (100 mg/m^2, 20 mg./m.2,1-5 mg/m^2)	Therapeutic equivalence (three cycles of BEP and four cycles of etoposide-cisplatin	[36]
Metastatic testicular cancer found in one man	Mediastinoscopy, biopsy and histological examination	PET scan	Specificity problem of FDG-PET scanning	[37]
Testicular seminoma stage II found in 126 men:	Serum tumor markers (AFP, HCG), Chest radiograph	Radiotherapy (25-45 Gy), etoposide, cisplatin	Treatment for stage IIC seminoma.	[38]
NSGCT in 438 men:	Serum tumor markers (STMs; AFP and bHCG)	Therapy of high-dose cisplatin plus autologous stem cell transplantation	Remove malignant metastases from NSGCT	[39]

Testicular tumor in a solitary testis of 7 men:	HIFU	Testicular profilaxis irradiation (18 to 20 Gy)	Ablating testicular cancer by transcutaneous HIFU.	[40]
NSGCT clinical stage I found in 44 men	Clinical examination, serum tumor markers, chest x-ray, CT/ultrasound of abdomen, blood test, creatinine, AST, ALP	Orchiectomy, one cycle of BEP (20 mg/m^2,40 mg/m^2, 120 mg/m^2)	Decretion in risk of relapse in patients with NSGCT stage I with BEP treatment (one cycle)	[41]
38 men with early-stage I or II and low-risk disease in:	Serum analysis; liver/renal test; thorax, abdomen, and pelvis CT scan; lumbar puncture and bone marrow biopsy	CEOP14 and (CEOP14R) regime	Improves outcome of poorest prognosis in patients	[42]
Testicular carcinoma found in 51 men	Levels of sex hormone–binding globulin, testosterone and LH	Chemotherapy: 20 Gy and 16 Gy	16 Gy RT better preserving testosterone levels	[43]
CS1 NSGCT found in 232 men	CT (thorax, abdomen and pelvis), serum tumor markers	CVB (20 mg/m^2, 0.15 mg/kg, 30 mg)	Survival for CS1 NSGCT patients treated	[44]
Stage I NSGCT found in 142 men	Histological examination	BEP (15 IU, 120 mg/m^2 and 40 mg/m2)	Effective and safe form of adjuvant therapy	[45]
Testicular seminomatous GCT in one man	Ultrasound, CT, chest radiographs, HCG and AFP levels	Four cycles of BEP salvage chemotherapy with four cycles of TIP, HDC, and ASCT	Complete remission (by imaging and serum biomarkers)	[46]
Testicular seminoma 10 men: stage I	CT scans	PBT (25.5 Gy)	Decreased and reduced risk of second cancers	[47]
TGCT found in 31 men	AFP elevated, bHCG without disease on imaging	Sunitinib (50 mg/day for 4 weeks)	Modest clinical activity	[48]
TGCT found in 157 men.	Serum tumor markers	PEB chemotherapy	Decrease in the estimated glomerular filtration rate	[49]
Testicular neoplasm in one man.	CT abdominal	PEB (4 cycles), orchiectomy	"Burned out" testicular neoplasm	[50]
Testicular seminoma found in 199 men:	Testicular ultra-sound and serum measurement AFP, bHCG, CT scan	Orchiectomy, radiotherapy	Excellent long-term prognosis	[51]
Testicular seminoma stage 1 found in 517 men.	Clinical examination, chest X-ray levels of AFP, bHCG and LDH.	Orchidectomy, carboplatin AUC-7	Carboplatin as safe and effective adjuvant treatment	[52]
YST found in 5 men	AST, ALP, LDH, AFP, bHCG levels, CT (brain, chest, and abdomen), technetium-99 bone	PVB and JEB regimen	JEB is sufficient as treatment option.	[53]

	scan			
SBT found in one man.	Ultrasonography, MRI	Orchiectomy	MRI could help to clinicians to differentiate SBT from other testis tumors	[54]
Primary adenocarcinoma of the rete-testis found in one man.	Immunohistochemical, PET-CT.	Orchiectomy, RPLND	PET-CT is useful detecting metastasis	[55]
Relapsed GCT and no curable options in 20 men	Histology, increase of AFP, bHCG.	Pembrolizumab (200 mg)	Pembrolizumab did not demonstrate clinical benefit	[56]
NSGCTT vascular invasion–positive stage 1 in 246 men	CT (chest, abdomen, pelvis), AFP, LDH and HCG.	Orchidectomy, PEB (30000 IU, 165 mg/m^2, 50 mg/m^2)	Cisplatin (BE500P) was safe in use	[57]
SEMs (53) non-seminomatous (37) tumours.	CT (chest, abdomen), ultrasonography, levels of hCGb, AFP, hPLAP and LDH in the cubital vein	24.5% of peripheral vein bHCG was positive (+), vs 84.6% bHCG (+) in the testicular vein.	Testicular tumor markers are more frequently positive in the spermatic vein as compared to the cubital vein	[58]
